# Effect of peer-led health professional-supported intervention on cardiovascular disease risk reduction among industrial workers of Pokhara, Nepal: A quasi-experimental study

**DOI:** 10.1371/journal.pgph.0004639

**Published:** 2025-08-12

**Authors:** Bibash Rana, Chiranjivi Adhikari, Anil Khanal, Muskan Pudasainee, Shiksha Adhikari, Bishnu Kumari Khattri, Damaru Prasad Paneru

**Affiliations:** 1 School of Health and Allied Sciences, Pokhara University, Pokhara, Gandaki Province, Nepal; 2 Indian Institute of Public Health-Gandhinagar (IIPHG), Gandhinagar, Gujarat, India; 3 Sanjeevani College of Medical Sciences, Purbanchal University, Butwal, Nepal; Universiti Malaya, MALAYSIA

## Abstract

Cardiovascular diseases (CVDs) are a leading cause of morbidity and mortality globally, posing significant health risks, particularly among industrial workers. Worksite-based interventions show promise for cardiovascular health but are limited in Nepal due to resource constraints, poor monitoring, and low participation. This study aimed to evaluate the effectiveness of a peer-led health professional-supported (PLHPS) intervention in reducing CVD risk factors among industrial workers in Pokhara, Nepal.A quasi-experimental study was conducted for eight weeks involving 129 industrial workers, divided into an intervention group (IG = 67) and a control group (CG = 62). The intervention group received a PLHPS intervention, which included interactive educational session, motivational interviewing, peer facilitation and monitoring while the control group did not receive any intervention. The comparison was made through the Mann-Whitney U test between the intervention and control groups on the Fuster-BEWAT score and test was applied by looking up on the data distribution. The effectiveness of the intervention was measured using components of the Fuster-BEWAT Score (FBS). Data were collected from both groups by post-test only through the Solstice application. With medium effects, the intervention group’s participants reported considerably increased intakes of fruits and vegetables (U = 2854, p < 0.001, r = 0.382) and changes in quitting tobacco (U = 2863, p < 0.01, r = 0.384). With a minor effect (U = 2423.5, p = 0.043, r = 0.178), the intervention group’s overall FBS differed significantly from that of control group. However, no significant differences were observed between the groups in terms of blood pressure, exercise, and weight (p’s > 0.05).The eight-week intervention for industrial workers is effective in initiating dietary and tobacco cessation behaviors but needs further reconfirmation after six months. Longer intervention periods and individualized intervention are needed for long-term effects and sustainable behavior change.

## Introduction

Cardiovascular diseases (CVDs) are a leading cause of mortality globally, with industrial workers particularly at risk due to lifestyle-related factors such as physical inactivity, inadequate consumption of fruits and vegetables, tobacco use, high blood pressure, and overweight and obesity [1–4]. Worksite-based interventions such as CVD education, tobacco cessation and prevention, physical activity, alimentation etc. are promising health promotion tools and have exhibited significant improvements in cardiovascular risk factor profiles [5,6]. In Nepal, even though such interventions are limited, a setting-based protocol proposing such an intervention has been hypothesized to be effective [7]. In addition, those programs are also hindered by a lack of human resources, insufficient monitoring, and low stakeholder participation [7,8]. In the meantime, a health partner (HP) intervention significantly improved cardio metabolic risk profile and cardiovascular health metrics among the risk population [9–13]. Moreover, involving peer leaders in addition to health professionals in the intervention resulted in significant improvements in common cardiovascular disease (CVD)-related behaviors, including blood pressure, exercise, weight control, diet, and tobacco cessation [14–16]. These behaviors are indexed as the Fuster-BEWAT Score (FBS), which is a simplified tool that does not require any laboratory investigations for assessing the CVD risks [17,18]. The Fuster-BEWAT Score (FBS) was chosen over the Ideal Cardiovascular Health Score (ICHS) which includes laboratory-dependent variables such as blood glucose and cholesterol, whereas FBS uses non-invasive, behavior-oriented metrics like blood pressure, exercise, weight, alimentation, and tobacco use, making it the first option in settings where the laboratory resources are limited, along with that comparable performance with ICHS in predicting subclinical atherosclerosis among low-risk individuals, validating its utility as a practical and reliable tool for assessing cardiovascular health [18]. Despite the global success of worksite-based interventions for CVD prevention, in Nepal there is lack of such type of structured programs targeting industrial workers. Additionally, no studies in Nepal have assessed the impact of peer-led models in CVD interventions, despite their proven success elsewhere. Moreover, culturally tailored interventions integrating peer leadership and health professional support are lacking, highlighting the need for a context-specific approach. The novelty of this study lies in its hybrid intervention design—Peer-led and Health Professional-supported (PLHPS)—which combines the social influence of peer facilitators with structured guidance from trained professionals. Our main motivation was to develop a practical, scalable, and contextually tailored intervention model to address preventable cardiovascular risk factors among an underserved workforce. So, this study aimed to develop and evaluate the effectiveness of PLHPS intervention in reducing CVD risk factors in an industrial setting. Based on existing evidence and formative findings, we hypothesized that the PLHPS intervention would lead to significant improvements in cardiovascular health behaviors, compared to control group among industrial workers.

## Methods and materials

### Study design, participants and sampling

**Fig 1:**
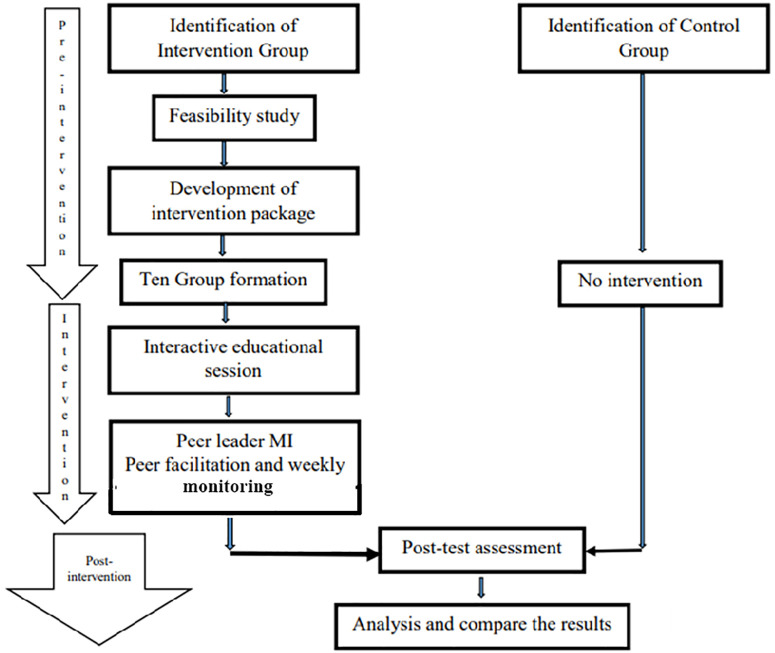
Study flow diagram.

A post-test-only quasi-experimental study was carried out for an eight-week period between the recruitment periods. Here in this study, we had used quasi-experimental design instead of RCTs, which are logistically feasible, and this design allows for real-world implementation, respects workplace structures, and maintains validity while being more feasible and ethically appropriate. The study was started on 28th April 2024 and ended on 23rd June 2024, whereas the participants were recruited between 28th April to 3rd May, which took one week for recruitment; likewise, immediately after the recruitment, the intervention period was started, and the post-test was done between 16th May to 23rd June. While conducting the post-test, the first recruited participants were post-tested. Since participants were enrolled in intervention on the day of recruitment, after completing an eight-week intervention, a post-test was conducted among the intervention group and then among the control group. Although the intended intervention period was 8 weeks, logistical constraints led to a slightly shorter duration for the intervention period. Likewise, due to the resource constraints and workers’ schedule hindered for pre-test, hence we were able to make post-test only design (Fig 1).

This study was designed assuming superiority to assess whether the PLHPS intervention would result in better cardiovascular health behaviors compared to no intervention (control group). Sample size was calculated using the effect size (d) as 0.77; the value was taken from a study using peer group–based intervention on cardiovascular risk factors using BEWAT Score [16]. Similarly, the standard deviation (σ) as 1.35 which was changed on BMI, taken from the study using health partner intervention [19]. Assuming an anticipated dropout rate (r) of 20% which was assumption for the similar settings, with values of α, Z_α_ and Z_β_ as 0.05 (two-tailed), 1.96 and 0.84 (80% power), respectively, which yielded a sample size of 62 in intervention (IG) and control group (CG) each. we enrolled 67 and 62 in the IG and CG, respectively, with a total sample of 129.

The participants were industrial workers from Pokhara Metropolitan City, Nepal. Employees >35 years of age who had been working in different industries located in Pokhara Metropolitan City at any position, such as managerial level or laborer, were recruited in the study. Similarly, pregnant women, mentally retarded and bed-ridden patients were excluded from the study. Firstly, industries were selected purposefully, and then, from the selected industries, participants were also selected purposefully from the worker list of each industry based on the inclusion criteria. Likewise, ten different industries located nearby the industrial area of Pokhara-14 were selected purposefully for the control group based on the inclusion criteria. While randomization was not possible, socio-demographic characteristics such as age, gender, education, and job type were assessed at post-test to ensure comparability between groups. A chi-square test was performed to examine any significant differences between the groups on these variables. This approach was adopted to reduce systematic differences and potential biases between the intervention and control groups.

### Pre-intervention phase

**Fig 2:**
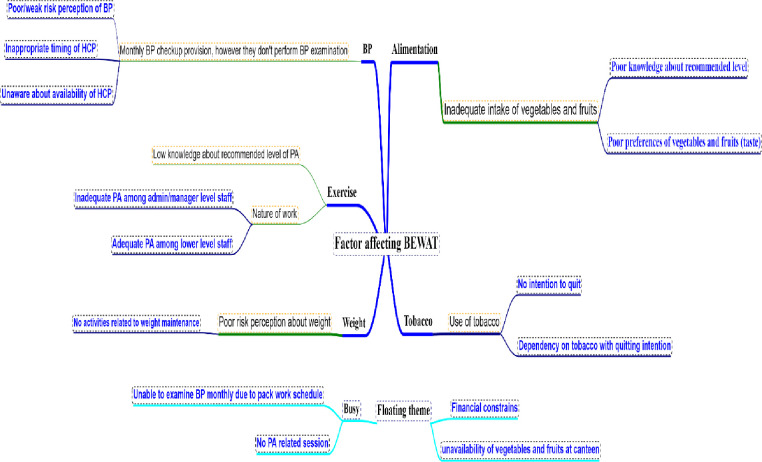
Factors affecting BEWAT components among industrial workers.

Four key informant interviews (KIIs), two in-depth interviews (IDIs), and two focused group discussions (FGDs) were conducted for the feasibility and formative studies. KII was conducted among the managerial and administrative staff of different industries to explore the intervention modality. Similarly, IDI and FGD were conducted among ground-level industrial workers. Deductive and inductive approaches were applied to develop intervention strategies and components (Fig 2). To ensure contextual relevance and acceptability of the intervention, qualitative methods were employed in the pre-intervention phase. Key Informant Interviews (KIIs) with managerial and administrative staff provided insights into workplace policies, logistical feasibility, and potential facilitators and barriers to program implementation. In-depth Interviews (IDIs) with individual industrial workers captured personal health behaviors, motivational factors, and individual challenges related to cardiovascular risk. Focus Group Discussions (FGDs) encouraged collective reflection and revealed shared perceptions, social norms, and group dynamics that influence health behaviors. Using this combination of qualitative methods allowed for a comprehensive understanding of both institutional and individual perspectives, which was essential in developing a culturally sensitive and practically feasible intervention package tailored to the needs of industrial workers. Qualitative data obtained from KIIs, FGDs and IDIs were intelligent verbatim transcribed and then thematic analysis was carried out manually. These transcriptions were reviewed multiple times to identify recurring ideas and patterns. Both deductive and inductive strategies were applied—deductive to align with pre-identified constructs such as BEWAT-related behaviors, and inductive to capture new, context-specific insights. Though the absence of full transcription may limit the depth of analysis, this pragmatic approach enabled rapid theme identification that was directly used to design the intervention package tailored to the industrial setting.

Based on the feasibility findings, an intervention package was planned. The feasibility phase refers to the formative qualitative assessment conducted through key informant interviews, in-depth interviews, and focus group discussions to explore workplace readiness, logistical barriers, and cultural acceptability of the intervention. An intervention package was developed based on feasibility findings, involving a literature review, pretesting, and feedback from colleagues. Their feedback focused on the clarity, cultural appropriateness, and consistency of intervention materials such as lesson plans and brochures. This feedback was used to revise the language, visual layout, and delivery strategy to ensure alignment with the industrial context. Although a baseline quantitative pretest was not conducted due to logistical limitations, the qualitative pre-intervention assessments served as the foundation for contextualizing and tailoring the intervention content and delivery. Peer leader facilitation brochures, health professional-supported brochures, and lesson plans were also developed through an extensive literature review, and feedback was collected for the consistency and relevancy of the intervention package.

### Intervention

For intervention (IG), ten groups were formed in nine different industries, with 4–14 members in each group, which was based on the availability and permission of workers to participate in the study from each industry. The IG received a PLHPS intervention package that included educational sessions and monitoring for both peer leaders and participants, whereas motivational interviewing (MI) and peer facilitation aided with telephonic dialogue conversations were carried out for peer leaders. We conducted a one-hour interactive educational session in each group with a lesson plan in the local language (S1 Text). These sessions were supported with distribution of brochure in the local language to each participant (S2 Text). During the interactive session, peer leaders were selected using a participatory and democratic process based on predefined criteria to ensure their potential to influence and motivate peers. Sociometry was assessed through a simple nomination technique, where participants were asked to identify individuals in their group whom they trusted, frequently interacted with, and considered capable of providing health-related guidance. The individuals receiving the highest number of nominations were considered to have high sociometric standing. Literacy was assessed through brief oral and written screening during the session, including the ability to read and explain basic health messages from the intervention brochure. Only those who demonstrated sufficient literacy and willingness to participate were considered for the peer leader role. All selected peer leaders were employed in their respective industries and were familiar with their peers’ routines and challenges.

Peer leader MI was conducted by health professionals in the 2nd and 5th weeks by visiting each peer leader at their working environment for enhancement of healthy behaviors, emphasizing the BEWAT component, and monitoring the healthy behaviors of their group members. Each interview lasted for 15–20 minutes. A peer leader brochure was disseminated to all of the peer leaders to enhance their understanding of the contents during the first MI.

Similarly, to ensure uniformity and fidelity in the delivery of the intervention, a structured monitoring plan was implemented. Health professionals were provided with a facilitation brochure that outlined standardized content and procedures focused on BEWAT components, ensuring consistent messaging across all sessions. Peer leader facilitation and monitoring were done each week from the 1st to the 7th week after the interactive educational session. Weekly monitoring of peer leaders was conducted through scheduled phone calls, in which health professionals used a semi-structured checklist to track peer leader engagement, clarity of message delivery, behavioral monitoring of group members, and problem-solving strategies used. During the second and fifth weeks, health professionals conducted in-person motivational interviewing sessions with peer leaders, using a structured guide to reinforce intervention and assess the implementation quality. Any discrepancies or challenges identified were addressed through tailored feedback and reorientation, promoting fidelity to the intervention model across all groups.

The control group (CG) did not receive any intervention during the study period to allow for a valid comparison of outcomes and to assess the net effect of the PLHPS intervention in a real-world setting. This approach reflects the current situation in many Nepali industrial settings, where structured health promotion activities are largely absent. To address ethical considerations, all participants were fully informed about the group allocation and study design. Additionally, the CG participants were assured of receiving the same health education materials and key messages after the completion of the post-test, ensuring fairness and minimizing ethical concerns. After the eight weeks of interactive educational sessions, we conducted post-test assessments in both groups.

### Data management and analysis

Post-tests were conducted among both the interventional and control groups after the eight weeks of intervention implementation. To ensure data accuracy and reliability, several quality assurance measures were implemented throughout the data collection process. Data collected via the Solstice application were reviewed daily by the research team to check for completeness, consistency, and entry errors. Any discrepancies were verified through direct follow-up with data collectors. Skip logic and mandatory fields were pre-set in the digital form to minimize missing or invalid responses. The data collection devices were password-protected, and only authorized team members had access to the records. Updated data was downloaded on a laptop; likewise, data was exported in Microsoft Excel 2013 and transferred to Statistical Package for Social Science (SPSS) version 22 for analysis (S1 Data). Along with that, the devices used for data collection were carefully handled and ensured that the entered data was complete and correct.

### Descriptive statistics

For the analysis of the posttest assessment, frequencies, percentages, medians, quartiles, minimums, and maximums were used to summarize the data after the normality test of the data. The overall Fuster-BEWAT score in the ideal metric was presented as the median score.

### Inferential statistics

The chi-square test was used to assess the significance between IG and CG based on the socio-demographic variables. Prior to conducting inferential analyses, data distribution was assessed for normality. The results indicated that several continuous variables, including the Fuster-BEWAT Score (FBS) components, were not normally distributed. Consequently, nonparametric tests were deemed more appropriate. The Mann-Whitney U test was used to compare differences between the intervention and control groups, as it does not assume a normal distribution and is suitable for ordinal or non-normally distributed continuous variables. This test allowed for robust analysis of group differences while accounting for the distributional characteristics of the dataset. Likewise, effect size (r) was calculated based on the Z value of the Mann-Whitney U test to assess the effect size between the groups.

### Ethical consideration

Ethical approval with reference number 137/2080/81 was taken for this study from the Institutional Review Committee of Pokhara University based on its protocol and format. Written and verbal inform consents were taken from each respondent before the data collection, along with those ethical aspects mentioned in the Helsinki declaration. Data collection was conducted in Nepali language to ensure participants’ understanding. A short counseling was given to the participants with high and moderate CVD risks.

## Results

### Descriptive statistics

#### Socio-demographic characteristics.

A total of 129 industrial workers were enrolled in the study following eligibility screening and consent. Based on workplace assignment, 67 participants were allocated to the intervention group and 62 to the control group. No participants withdrew or were lost to follow-up during the intervention period. All 129 participants completed the post-test and were included in the final analysis.

Our study showed that the median age of participant was 42 years. The majority of respondents were male, with 83.7% being male, whereas in the intervention, about three-quarters (74.62%), and 93.5% in the control group. About half of the participants (48.1%) were Bhramin/Chhetri ethnic group, whereas not a single individual was identified as Muslim in intervention group, but 16.1% were in control group. Primary school and literate categories were higher in both intervention and control groups (41.8% and 38.7%), respectively. Nearly one-quarter (23.9%) completed a high school certificate in IG, while middle school and high school certificate categories were higher in CG (24.2%) and 22.6%, respectively. The median monthly family income was Rs. 43891. Upper lower and lower middle socio-economic classes covered a higher portion in both group (45% and 42%, respectively) (Table 1).

**Table 1:** Socio-demographic characteristics.

Variables	Overall (n = 129)		Intervention group (n = 67)		Control Group (n = 62)	
	Frequency	%	Frequency	%	Frequency	%
**Age**						
35-39	48	37.2	12	17.9	36	58.1
40-44	29	22.5	11	16.4	18	29.0
45-49	17	13.2	13	19.4	4	6.5
50-54	20	15.5	18	26.9	2	3.2
≥55	15	11.6	13	19.4	2	3.2
*M*_*d*_ *(Q*_*1*_ * ~ Q*_*3*_ *)[Min ~ Max]*			*42(38 ~ 50)[36 ~ 68]*			
**Gender**						
Male	108	83.7	50	74.62	58	93.5
Female	21	16.3	17	25.37	4	6.5
**Ethnicity**						
Brahmin/Chhetri	62	48.1	39	58.2	23	37.1
Dalit	7	5.4	2	3.0	5	8.1
Janajati	41	31.8	23	34.3	18	29.0
Madhesi	9	7.0	3	4.5	6	9.7
Muslim	10	7.8	0	0	10	16.1
**Education**						
^1^Primary School or literate	52	40.3	28	41.8	24	38.7
Middle School certificate	23	17.8	8	11.9	15	24.2
High school certificate	30	23.3	16	23.9	14	22.6
Higher secondary certificate	16	12.4	9	13.4	7	11.3
^2^Graduate, post-graduate and professional degree	8	6.2	6	9.0	2	3.2
**Income**						
^@^ ≤ 14550	11	8.5	20	29.9	11	17.7
14551-24350	37	28.7	19	28.4	17	27.4
24351-36550	40	31.0	5	7.5	21	33.9
36551-48750	11	8.5	23	34.3	6	9.7
^@@^ ≥ 48751	30	23.3	20	29.9	7	11.3
*M*_*d*_*(Q*_*1*_* ~ Q*_*3*_*)[Min ~ Max]*			*25000,(15000,31250)[5000 ~ 700000]*			
**Occupation**						
^3^Semi-skilled worker	46	35.7	31	46.3	15	24.2
Skilled worker	56	43.4	19	28.4	37	59.7
Arithmetic skill worker	13	10.1	8	11.9	5	8.1
^4^Sem-professional and professional worker	14	10.9	9	13.4	5	8.1
Lower	0	0	0	0	0	0
Upper lower	58	45	26	38.8	32	51.6
Lower middle	55	42	30	44.8	25	40.3
Upper middle	16	12.4	11	16.4	5	8.1
Upper	0	0	0	0	0	0

^1^ Illiterate merged into primary or literate group; ^2^Post graduate and professional degree merged into graduate degree group;^@^ < 4850 added to upper group and categorized as < 14550; ^@@^ > 97451 group also merged its upper group and categorized as ≥48751; ^3^ Unskilled added in semi-skilled group in IG; ^4^professional worker merged into Sem-professional group.

### Inferential statistics

#### Comparison between the intervention and control group.

The findings revealed that there were significant differences in age of participants (p < 0.001^*^), gender (p = 0.004^*^), ethnicity (p = 0.016^*^), income (p = 0.001^*^) and occupation (p = 0.005^*^) between the intervention and control group. While other two remaining variables education and socio-economic class between the intervention and control group were not significantly differ (Table 2).

**Table 2:** Comparison between the intervention and control group.

Variable	Intervention group	Control group	Chi-square statistic	p value
**Age**	(n = 67)	(n = 62)		
35-39	12	36	38.477	**<0.001** ^*^
40-44	11	18		
^1^ ≥ 45	44	8		
**Gender**				
Male	50	58	8.459	**0.004** ^*^
Female	17	4		
**Ethnicity**				
Brahmin/Chhetri	39	23	5.750	**0.016** ^*^
^2^Others	28	39		
**Education**				
^3^Primary School or literate	28	24	4.635	0.327
Middle School certificate	8	15		
High school certificate	16	14		
Higher secondary certificate	9	7		
^4^Graduate, post-graduate and professional degree	6	2		
**Income**				
^5^ ≤ 14550	20	11	19.803	**0.001** ^*^
14551-24350	19	17		
24351-36550	5	21		
36551-48750	23	6		
^6^ ≥ 48751	20	7		
**Occupation**				
^@^Semi-skilled worker	31	15	13.012	0.005*
Skilled worker	19	37		
Arithmetic skill worker	8	5		
^@@^Semi-professional and professional worker	9	5		
**Socio-economic class**				
^$^Upper lower	26	32	3.136	0.208
Lower middle	30	25		
^$$^Upper middle	11	5		

*p-value significance at α level of 0.05%; ^1^more than 45 years participants categorized into > 45 age group (highest age of participants was 68 years); ² Includes Dalit, Janajati, Madhesi, and Muslim ethnic groups; ^3^Illiterate merged into primary or literate group; ^4^Post graduate and professional degree merged into graduate degree group; ^5^ < 4850 added to upper group and categorized as < 14550, ^6^ > 97451 group also merged its upper group and categorized as ≥48751; ^@^Unskilled added in semi-skilled group in IG; ^@@^professional worker merged into Sem-professional group; ^$^Lower socioeconomic class merged intro upper lower; ^$$^Upper socioeconomic class merged into upper middle.

### Fuster-BEWAT score and effectiveness

The intervention group (IG) and control group (CG) were compared across the five components of the Fuster-BEWAT Score (FBS): blood pressure (B), exercise (E), weight (W), alimentation (A), and tobacco use (T).

#### Blood pressure (BP).

The study found that over one-third of respondents had normal blood pressure in both groups (IG: 38.8%; CG: 35.5%), with a significant portion having elevated blood pressure. However, there was no significant difference between the IG and CG groups in BP with negligible effect size (U = 2162, p = 0.672, r = 0.037) (Table 3).

**Table 3:** Fuster-BEWAT Score and effectiveness.

BEWAT	Score	Total		Intervention		Control		U-statistic, p-value	Standard normal variate (Z)	Effect size (r)
		n = 129	%	n = 67	%	n = 62	%			
BP	0 (Stage-2 = SBP > 140 and/or DBP > 90 mm Hg)	33	25.6	17	25.4	16	25.8	2162^b^,.672	0.423	0.037
	1 (Stage-1= (SBP 130–139 and/or DBP 85–89 mm Hg)	17	13.2	9	13.4	8	12.9			
	2 (Elevated= (SBP 120–129 and/or DBP 80–84 mm Hg)	31	24.0	15	22.4	16	25.8			
	3 (Normal= (SBP < 120 and DBP < 80 mm Hg)	48	37.2	26	38.8	22	35.5			
*M*_*d*_*(Q*_*1*_* ~ Q*_*3*_)				*2(0 ~ 3)*		*2(0 ~ 3)*				
Exercise	0 (<10 min moderate to vigorous activity min/week)	26	20.2	8	11.9	18	29	2168^b^,.628	0.485	0.042
	1 (<75 min moderate to vigorous activity min/week)	20	15.5	14	20.9	6	9.7			
	2 (75–149 min moderate to vigorous activity min/week)	7	5.4	7	10.4	0	0			
	3 (> 150 min moderate to vigorous activity min/week	76	58.9	38	56.7	38	61.3			
*M*_*d*_*(Q*_*1*_* ~ Q*_*3*_)				*3,(0 ~ 3)*		*3,(0 ~ 3)*				
Weight	0 (Obese= > 30 kg/ m^2^)	12	9.3	5	7.5	7	11.3	2082^b^,.978	0.027	0.002
	1 (Overweight = 25 to <30 kg/ m^2^)	41	31.8	23	34.3	18	29			
	3 (Normal= < 25 kg/m^2^)	76	58.9	39	58.2	37	59.7			
*M*_*d*_*(Q*_*1*_* ~ Q*_*3*_)				*3,(1 ~ 3)*		*3,(1 ~ 3)*				
Alimentation	0 (<1 serving of fruit/vegetables daily)	0	0	0	0	0	0	2854^b^, < 0.001*	4.347	0.382
	1 (1–2 serving of fruit/vegetables daily)	45	34.9	12	17.9	33	53.2			
	2 (3–4 serving of fruit/vegetables daily)	81	62.8	52	77.6	29	46.8			
	3 (>4 serving of fruit/vegetables daily)	3	2.3	3	4.5	0	0			
*M*_*d*_*(Q*_*1*_* ~ Q*_*3*_)				*2,(2 ~ 2)*		*1,(1 ~ 2)*				
Tobacco	0 (Current smoker)	31	24	3	4.5	28	45.2	2863^b^, < 0.01*	4.372	0.384
	1 (Former smoker)	15	11.6	11	16.4	4	6.5			
	3 (Never)	83	64.3	53	79.1	30	48.4			
*M*_*d*_*(Q*_*1*_* ~ Q*_*3*_)				*3,(3 ~ 3)*		*1,(0 ~ 3)*				
Number of ideal metrics	1	12	9.3	4	6.0	8	12.9	2518^b^, 0.029*	2.188	0.192
	2	50	38.8	23	34.3	27	43.5			
	3	40	31.0	22	32.8	18	29.0			
	4	26	20.2	17	25.4	9	14.5			
	5	1	.8	1	1.5	0	0			
*M*_*d*_*(Q*_*1*_* ~ Q*_*3*_)				*3,(2 ~ 4)*		*2,(2 ~ 3)*				
FBS	1 (Poor)	12	9.3	4	6.0	8	12.9	2423.5^b^, 0.043*	2.025	0.178
	2 (Intermediate)	90	69.8	45	67.2	45	72.6			
	3 (Ideal)	27	20.9	18	26.9	9	14.5			

^b^Mann-Whitney U test statistic; *p-value significance at α *level of 0.05%.*

#### Exercise (E).

Both groups reported similar physical activity patterns, with over half exceeding 150 minutes of moderate-to-vigorous activity per week (IG: 56.7%; CG: 61.3%). The difference was not statistically significant (U = 2168, p = 0.628, r = 0.042) (Table 3).

#### Weight (W).

In both groups, the majority of respondents were identified as having a having a normal BMI (IG: 58.2% and CG: 59.7%). Similarly, no significant difference were observed with trivial effect on weight (p = 0.978, r = 0.002) (Table 3).

#### Alimentation (A).

A significant difference was observed in daily fruit and vegetable (FV) consumption (U = 2854, p < 0.001, r = 0.382). In the IG, 77.6% consumed 3–4 servings daily, and 4.5% consumed more than 4 servings. In contrast, 53.2% of the CG consumed only 1–2 servings, and none reported more than 4 servings.

#### Tobacco (T).

As expected, a significant difference was observed between IC and CG with a p value <0.01 in tobacco cessation, along with a medium effect (U = 2863, p < 0.01, r = 0.384). The proportion of never-smokers was notably higher in the IG (79.1%) compared to the CG (48.4%), while current smokers were significantly more common in the CG (45.2%) versus the IG (4.5%).

#### Overall fuster-BEWAT score (FBS).

After the intervention, the intervention group shows significant differences in the overall FBS in comparison to the control, along with small effect (U = 2423.5, p = 0.043, r = 0.178). A higher proportion of IG participants achieved ideal cardiovascular health scores (26.9%) compared to the CG (14.5%).

These results support the hypothesis that the PLHPS intervention leads to improvements in alimentation (fruit and vegetable consumption) and tobacco cessation behaviors compared to the control group.

## Discussion

This study found that the peer-led health professional-supported intervention (PLHPS) was effective in improving two key modifiable cardiovascular risk behaviors—fruit and vegetable consumption and tobacco cessation—among industrial workers. These are among the most critical behaviors associated with cardiovascular health, and the observed medium effect sizes (r = 0.382 for alimentation and r = 0.384 for tobacco use) reflect meaningful health behavior change. While changes in blood pressure, weight, and physical activity were not statistically significant, the overall Fuster-BEWAT Score (FBS) still showed a small but significant improvement (U = 2423.5, p = 0.043, r = 0.178) in eight week. The findings of this study support our initial hypothesis that the PLHPS intervention would significantly improve dietary behavior and tobacco cessation among industrial workers. While the hypothesis was supported for alimentation and tobacco cessation behaviors, it was not supported for physical activity, weight, or blood pressure, likely due to the short duration of the intervention and its cognitive (rather than behavioral) focus. Here we discuss the effectiveness of the intervention in each and aggregate BEWAT, and possibility of sustainable maintenance of those behaviors even after two months. For this, we highlight the discussion, mainly with stage-based theories and models, and focusing setting-based approach.

The significant improvement in the overall FBS observed in our study is partially consistent with the study results carried out by Gomez-Pardo et al. in the Fifty-fifty Program [16], which reported substantial improvements in FBS after a year-long peer-led intervention. This similarity might be due to the study conducted on adults with similar risk factors, such as obesity and hypertension, along with peer-led interventions. Additionally, in our study, the culturally tailored content, peer selection based on trust and sociometric standing, and weekly reinforcement through health professional support likely contributed to the observed behavioral shifts. In contrast, while our study showed improvements in the overall FBS, the short duration of eight weeks might have limited the extent of changes in components such as blood pressure, weight, and exercise, which are similarly observed even in a longer (12-month) duration intervention— the Grenada Heart Project-Community Health ActioN to EncouraGe healthy BEhaviors (GHP-CHANGE)— carried out by Latina et al., which showed that despite being feasible, peer-led lifestyle intervention is not effective in improving FBS components [19]. Failure to achieve these components may be indirectly associated and mediated with lack of self-efficacy, as in case of self-reporting and decisional balance of one of the FBS components, the physical activity, as explored in Pinto et al.’s study [20]. Similarly, lacking perceived behavioral control, and thereby, behavioral intention may also hinder to achieve the FBS targets [21].

A systematic review exhibited that worksite intervention using social support and peer strategies was effective in increasing FV daily serving consumption [22]. The observed increase in fruit and vegetable consumption in the intervention group is consistent with the findings of a faith-placed CVD risk-reduction intervention in rural Illinois [23] implemented. The study reported significantly higher vegetable and combined fruit/vegetable consumption among participants who engaged in both the Heart Smart for Women (HSFW) and Heart Smart Maintenance (HSM) programs compared to those in the HSM-only group (p = 0.007 and p = 0.01, respectively). The similarities in dietary improvements between our studies can be attributed to group-based intervention designs, continuous support, and culturally relevant approaches in the respective context, such as leader selection and mobilization to facilitate their group members in achieving healthy behaviors. In our study, the combination of interactive educational sessions, motivational interviewing, and continuous peer facilitation created a supportive environment that likely reinforced healthy eating behaviors. Similarly, in the previous use of a combination of weekly and monthly sessions provided continuous reinforcement in changing fruit and vegetable consumption but could not reinforce for physical activity [23]. Similarly, a workplace-based 2-year intervention in promoting the fruit and vegetable (FV) consumption exhibited an increase in FV consumption, whereas the study used the cues to environmental change (offer FV in companies’ cafeterias) and educational sessions targeting individuals [24]. Additionally, we used peer strategies and continued support to achieve healthy behaviors regarding FV daily serving consumption, which might be the major reason for the significant improvement in FV consumption. Along with that, some of the respective industries in Pokhara offered FV lunch and snacks for their workers.

A systematic review exhibited that nicotine replacement therapy, information, and behavioral skills training interventions through peer strategies were effective in tobacco cessation even after the five-week intervention. However, the review have not discussed the sustainability of tobacco cessation or the long-term effects of peer support interventions [14]. Similarly, the intervention’s impact, as in ours, aligns with previous peer group-based intervention, the Fifty-fifty program [16], which emphasized the effectiveness of peer-led strategies in modifying health behaviors. Similarly, another BEWAT component, reduction in tobacco use, was found significant in the Health partner intervention [9]. The use of motivational interviewing with peer leaders may have played a key role, offering individualized support and reinforcing commitment to change, along with a participatory peer selection process that likely enhanced the credibility and influence of peer leaders within their groups, furthering behavioral shifts.

Although blood pressure (BP) component was not found significant, in contrast, a systematic review synthesized by Dickinson et al. showed that specific interventions emphasized on improved diet and aerobic exercise, alcohol and salt restriction significantly reduced blood pressure by 5 mmHg and 4.6 mmHg, respectively by 8–260 weeks [25]. The difference between our findings and those of Dickinson et al. might be attributed to several factors, such as the fact that their study participants had elevated blood pressure at baseline, whereas our study included participants with and without multiple risk factors such as tobacco use, inadequate use of fruit and vegetables, and high blood pressure, which means participants may not have high blood pressure but may present other risk factors. Moreover, in the review, interventions included a weekly schedule of three to five supervised sessions of aerobic exercise, together with diets targeting weight reduction, encouraging intake of fruit and vegetables, reducing fat intake, restriction of alcohol and increasing carbohydrate intake. Our study offered knowledge enhancement through cognitive intervention through the mobilization of peer leaders’ and health professionals’ support rather than behavioral intervention. Furthermore, a meta-analysis as in conducted among the adult participants with elevated blood pressure showed that healthcare professional-led interventions on lifestyle modifications resulted in a mean systolic blood pressure reduction of -4.41 mmHg and a diastolic reduction of -1.66 mmHg [12]. Their review emphasized that individualized interventions on dietary approaches to stop hypertension (DASH), tailored interventions, physical activity, smoking cessation, and adherence with medication with at least six-month intervention duration led by healthcare professionals result in significant blood pressure reductions and improve control rates among hypertensive patients. This suggests that targeting to individuals with elevated blood pressure, along with lifestyle modification behavioral intervention in six months period can significantly reduce the blood pressure. For the difference in our result, the possible major attributions could be the duration, individualized interventions and study participants.

Similarly, the result presented here conflicts with previous studies [8,13]. While our study didn’t show significant increase in physical activity, these prior studies using peer-led interventions with strong social support and a focus on self-efficacy, along with the intervention through cultural tailoring and frequent follow-ups for up to two years, likely contributed to their success. A potential reason for this discrepancy could be the lack of significant change in physical activity levels may reflect contextual barriers within the industrial setting, such as limited time or space for exercise, or lack of institutional support for active breaks. Our intervention emphasized cognitive strategies and behavioral support, rather than structured physical activity programming, which may explain the limited changes observed in exercise patterns. These findings suggest that our intervention needs to strengthen peer-led health professionals, be more tailored, and offer more social support to improve physical activity outcomes for participants, along with a longer duration of intervention and follow up for longer period.

A systematic review revealed that the weight management interventions by HCP are effective, particularly for women and adults, and most effective with at least a six-month intervention with minimum of six sessions though, they couldn’t achieve and maintain a healthy weight [26]. In the same line, a six-month intervention and assessed after 12 months showed that walking behavior and lipid reduction occurred though, BP, BMI and weight were not maintained [27]. These pieces of evidence suggest that experiential interventions by health professionals for overweight and obese individuals for at least six months are effective in weight reduction but not in weight maintenance. It seems plausible that cognitive interventions of only eight weeks, like ours, can reduce weight but not maintain it. The study aimed at nutrition and exercise-related interventions given to 1128 middle school students for obesity prevention could significantly reduce the BMI percentile [28]. A multi-strategy study combined with school-wide environmental changes, encouragement to eat healthy school cafeteria foods, and peer-led education interventions including motivational interviewing, including role play and a short film show, as an intervention method. However, our research did not show a significant difference in weight outcomes between the intervention and control groups in alimentation. This contrast could be due to several factors, including the shorter duration of our intervention, a smaller sample size, differences in intervention components, or variations in participant demographics and settings. The needs of the individual may vary, and our study emphasized the different topics covering multiple risks that may hinder the effectiveness of intervention in specific aspects. Similarly, the GHP-CHANGE trial demonstrated significant improvements in composite scores related to cardiovascular risk factors among adults, including BMI, in a low-income setting, highlighting the effectiveness of peer support strategies [19]. Our study did not find significant differences in weight outcomes between the intervention and control groups, diverging from the GHP-CHANGE trial that reported positive weight management results. The study offered an intense educational lecture series and at least three workshops to all participants, along with a peer leader who got additional training sessions on leadership and communication skills. The nature of the intervention and participants with CVD risk were similar to our study, despite that, one possible reason for the discrepancy could be shorter duration of our intervention compared to the longer periods (up to 12 months), which might have been insufficient to observe significant changes in weight. Additionally, other background characteristics of participants, like age (mean age 51.4 years) and sex (female 65.9%), were different in comparison to the study of Latina and colleagues (19).

Our study emphasized on the cognitive intervention, however findings of our study exhibited significant improvement in the alimentation and tobacco cessation component in the two months intervention period, which were behavioral outcomes. Individuals could change the behaviors and achieve the action stage even in a short period of time with such a cueing event that increases the perceived personal risk and self-efficacy, prompts strong emotional or affective responses, or that redefines social role or self-concepts [29]. However, these rapid progressions do also have high chance of relapses. There are inadequate studies and need of further more studies required to conduct emphasizing the long-term effects and sustainability of dietary changes and tobacco absenteeism behaviors [14,21,30] since it is unclear about the sustainability of lifestyle changes only with six-month intervention [30]. However, evidence indicates that even tiny improvements of risk factors such as diastolic blood pressure among the hyper- and normotensives can dramatically lower CVD risk mainly stroke and CHD [31]. Similarly, different tiers of government may collaborate with non-government organizations to improve CVD related preventive services in the country like Nepal [32].

### Strength and limitation of the study

A key strength of this study lies in setting-based design, which allowed for real-world application and high relevance to industrial workforces in similar contexts. Additionally, the use of the Fuster-BEWAT Score enabled non-invasive, scalable CVD risk assessment without laboratory data. Another strength is the integration of peer leadership, which provided culturally resonant support and facilitated implementation in the workplace by health professionals.

However, the study also has limitations. The absence of a pre-test assessment restricts the ability to measure change over time within individuals. Potential selection bias cannot be ruled out due to non-randomized group allocation, though the bias was tried to be minimized by selecting the participants from similar settings and age groups. Furthermore, the short duration of eight weeks and reliance on cognitive (rather than behavioral) strategies may have limited the impact on outcomes like blood pressure and weight.

### Conclusion

The peer-led health professional-supported eight-week intervention among industrial workers is medium effective (noticeable when carefully observed) in initiating alimentation and tobacco cessation behaviors, also implicating the overall Fuster-BEWAT score. However, this may need further reconfirmation at least after six-month assessment. Despite these encouraging results, the intervention did not yield significant changes in blood pressure, physical activity, or weight reduction. This may be attributed to the short duration of the intervention, the use of cognitive (rather than behaviorally intensive) strategies, and the absence of pre-test measurements due to workplace and resource constraints.

Overall, this study supports the feasibility and preliminary effectiveness of PLHPS interventions in industrial settings. Further research is needed to incorporate longer intervention periods, at least six months, to assess the sustainability of behavior change and the long-term effects of intervention in a more individualized way with greater emphasis on social support and self-efficacy to achieve significant improvements in BP, exercise, and weight among industrial workers.

## Supporting information

S1 Data Meta data. (XLSX)

S1 Text Lesson plan. (DOCX)

S2 Text Brochure. (DOCX)

## Abbreviations

BEWAT: B-Blood Pressure; E-Exercise; W-Weight; A-Alimentation; T-Tobacco
FBS: Fuster-BEWAT Score
MI: Motivational Interviewing
PLHPS: Peer-led Health Professional-supported
CVH: Cardiovascular Health
