# Supplementary material for: Effect of peer-led health professional-supported intervention on cardiovascular disease risk reduction among industrial workers of Pokhara, Nepal: A quasi-experimental study
Source: PLOS Glob Public Health. 2025 Aug 12;5(8):e0004639. doi: 10.1371/journal.pgph.0004639 (PMC12342279; doi:10.1371/journal.pgph.0004639)
Supplement: S1 Text — (DOCX) [file pgph.0004639.s002.docx]

**Effect of Peer-Led Health Professional-Supported Intervention on Cardiovascular Disease Risk Reduction among Industrial Workers of Pokhara, Nepal: A Quasi-Experimental Study**

**S1 Lesson plan**

विषय: हृदय रोग, यस्का कारकहरु र यसबाट बच्ने उपायहरु सम्बन्धि जानकारी

लक्षित वर्ग: औद्योगिक क्षेत्रका कर्मचारीहरु

शिक्षण बिधि: अन्तरक्रियात्मक छलफल, व्याख्यान

आवश्यक सामग्री: स्लाइड, जानकारी पुस्तिका, प्रोजेक्टर, ल्यापटप

समय: १ घण्टा

**उद्देश्यहरु:**

1. कार्यक्रमको महत्व बारेमा तथ्यांकको आधारमा जानकारी दिने
2. हृदय तथा रक्त नलीको रोगको छोटकरिमा परिचय दिने
3. रक्त नलिको संरचनाको बारेमा सामान्य परिचय दिने
4. मुख्य हृदय तथा रक्तनलिका रोगहरुका प्रकारहरुका बारेमा सामान्य जानकारी दिने
5. हृदय तथा रक्त नली रोगको मुख्य कारणहरुको बारेमा जानकारी दिने
6. जोखिमको कारकहरु बीचको आपसी अन्तर-समबन्धको बारेमा जानकारी दिने
7. हृदय तथा रक्त नली रोगको जोखिमहरुको बर्गिकरण
8. ब्यबहारहरुसंग सम्बन्धित मुख्य हृदय रोगको कारकहरुको बारेमा जानकारी दिने

८.१ उच्च रक्तचाप र अन्य जोखिम संगको सम्बन्धको बारेमा जानकारी दिने

८.२ सामनया बी एम आइ (तौल) र अधिक तौल तथा मोटोपनाको बारेमा जानकारी दिने, साथै मोटोपना र अन्य जोखिम संगको सम्बन्धको बारेमा जानकारी दिने

८.३ शारीरिक गतिबिधि र यस्को सिफारिश गरिएको स्तरको बारेमा जानकारी दिने

८.४ खानेकुराहरु (तरकारी तथा फलफूलहरु) तथा दैनिक सेवनको लागि सिफारिश गरिएको सर्भिङ्गको मात्राको बारेमा जानकारी दिने

८.५ धुम्रपानले मुटुमा गर्ने असरहरु तथा यस्को सेवन र अन्य जोखिम संगको सम्बन्ध

९. हृदय, रक्तनली तथा उक्त-रक्तचाप बढ्न बाट बच्ने उपाएहरु

**शिक्षण सिकाई क्रियाकलाप**

| क्र.स. | शिर्षक | शिक्षण सिकाई क्रियाकलापहरु | समय |
| --- | --- | --- | --- |
| १ | कार्यक्रमको महत्व | प्रश्न उत्तर, व्याख्यान | ५ मिनेट |
| २ | हृदय तथा रक्त नलीको रोगको परिचय | छलफल तथा व्याख्यान | ५ मिनेट |
| ३ | रक्त नलिको संरचना | व्याख्यान | ५ मिनेट |
| ४ | मुख्य हृदय तथा रक्तनलिका रोगहरुका प्रकार | प्रश्न उत्तर, छलफल तथा व्याख्यान | १० मिनेट |
| ५ | हृदय तथा रक्त नली रोगको मुख्य कारणहरु | प्रश्न उत्तर, छलफल तथा व्याख्यान |  |
| ६ | जोखिमको कारकहरु बीचको आपसी अन्तर सम्बन्ध | प्रश्न उत्तर, छलफल तथा व्याख्यान |  |
| ७ | हृदय तथा रक्त नली रोगको जोखिमहरुको बर्गिकरण | छलफल तथा व्याख्यान | ५ मिनेट |
| ८ | रक्तचाप | व्याख्यान | २० मिनेट |
| ९ | शारिरिक गतिबिधि | व्याख्यान |  |
| १० | तौल | व्याख्यान |  |
| ११ | खानपान (तरकारी र फलफूल) | व्याख्यान |  |
| १२ | धुम्रपान | व्याख्यान |  |
| १३ | हृदय, रक्तनली तथा उक्त-रक्तचाप बढ्न बाट बच्ने उपाएहरु | छलफल तथा व्याख्यान | १० मिनेट |

1. कार्यक्रमको महत्व बारेमा तथ्यांकको आधारमा सहभागीहरुलाई जानकारी दिने

- हृदय तथा रक्तनलि रोगका कारकहरु किन महत्वपूर्ण छन् भनि सहाभागीहरुलाइ सोध्ने, उहाहरुको उत्तरलाई सक्षेपमा प्रश्तुत गरि नेपालमा गरिएको अन्य अध्ययन तथा पोखराको औद्योगिक क्षेत्रमा गरिएको प्रारम्भिक अध्ययनको तथ्यांक अनुसार १०-१५ % कर्मचारिहरुमा यो रोगको जोखिम रहेको छ जसले गर्दा भविष्यमा ठुलो आर्थिक र स्वास्थ्यमा हानि हुन सक्छ त्यसैले यो कार्यक्रमको महत्व छ भनि सहभागीहरुलाई जानकारी गराई अनुभूति गराउने |

1. हृदय तथा रक्त नलीको रोग भनेको के हो ? सहभागीहरुसित छलफल गर्ने र सामान्य परिचय दिने |

- सहभागीहरुलाई हृदय तथा रक्त नलीको रोग भनेको के हो भनेर सोध्ने र उनीहरुको उत्तरको आधारमा यसको बारेमा प्रष्ट गराउने | र एथेरोस्क्लेरोसिसको बारेमा पनि जानकारि दिने |
- हृदय र रक्त नलीको रोग, जसलाई मुटु रोग पनि भनिन्छ, यसमा धेरै समस्याहरू समावेश छन्, जसमध्ये धेरै एथेरोस्क्लेरोसिससँग सम्बन्धित छन्।एथेरोस्क्लेरोसिस एक अवस्था हो जुन धमनीहरूको भित्तामा प्लाक भनिने पदार्थ निर्माण हुँदा विकसित हुन्छ। यो निर्माणले धमनीहरूलाई साँघुरो बनाउँछ, रगत प्रवाह गर्न गाह्रो बनाउँछ र अन्ततः यसले रगत प्रवाह रोक्न सक्छ। यसले हृदयघात वा मस्तिष्कघात निम्त्याउन सक्छ।

1. रक्त नलिको संरचनाको बारेमा चित्रबाट व्याख्यान गर्ने |

- सहभागीहरुलाई रक्तनलिको बारेमा सामान्य संरचनाको बारेमा बुझाउने जसले गर्दा उहाहरुलाई रक्तनलीको रोग लाग्दा रक्तनलिमा हुने असामान्य परिवर्तनको बारेमा जानकारी गराउने |

1. मुख्य हृदय तथा रक्तनलिका रोगहरुका प्रकार के के हुन् ? सहभागीहरुलाई सामान्य जानकारी दिने |

- सहभागीहरुलाई कुनै हृदय तथा रक्तनलिका रोगहरुका प्रकारको बारेमा सुनेको अथवा जानकारी भएनभएको सोधेर मुख्यतया हुने यी रोगहरुको प्रकारहरुको बारेमा सामान्य जानकारी दिने |

1. हृदय तथा रक्त नली रोगको मुख्य कारणहरु र जोखिम को कारकहरु बीचको आपसी अन्तर-सम्बन्ध चित्रमा देखाउने र छलफल गराई सक्षिपमा छलफलको जिस्ट भनेर चित्रले भन्नखोजेको कुरा प्रस्ट्याउने |
2. हृदय तथा रक्त नली रोगको जोखिमहरुको बर्गिकरणको बारेमा जानकारी गराउने र प्रस्ट गराउनलाइ सहभागीहरुसित बिभिन्न जोखिमहरू कुन लाइ कुन तहको हो भनि सोध्ने, जस्तै धुम्रपान सेवनलाइ कुन तहको जोखिम हो भनि सोध्ने, त्यस्तै अपर्याप्त शारीरिक गतिबिधि र तरकारी र फलफूलहरुको कम सेवन भएको व्यक्तिलाई कुन जोखिमको तहमा राख्ने भनि सोध्ने र छलफल गराई प्रस्ट गराउने |
3. मुख्य जोखिमहरूको बारेमा व्याख्यान गर्ने र सिफारिस गरे अनुसार शारीरिक गतिबिधि र खानेकुरा (तरकारी तथा फलफूलहरु) दैनिक ब्यबहारमा लागु गरेको नगरेको छलफल गर्ने | साथै मुख्य जोखिमहरुले गर्दा हृदय तथा रक्तनली रोग बीचको अन्तर सम्बन्धको बारेमा बिस्तृत जानकारी दिने |
4. हृदय, रक्तनली तथा उक्त-रक्तचाप बढ्न बाट बच्ने उपाएहरु के के हुन् भनेर छलफल गराउने र दैनिक रुप मा ति हरेक उपाएहरु अपनाउन प्रेरित गर्ने संगै सबैलाई मौखिक प्रतिबद्दता गराउने |
5. अन्त्यमा अन्तरक्रियात्मक कार्यक्रमको सारांस भन्ने र सहकर्मी नेतालाइ हरेक हप्ता आफ्नो स्वास्थ्य गतिबिधिहरुको निगरानीमा सहयोग पुर्याउन अनुरोध गर्ने |

**मूल्यांकन**

अन्त्यमा तलाका केहि प्रश्नहरु द्वारा सहभागीहरुको मूल्यांकन गरिने छ:

1. हृदय तथा रक्तनलीहरु को रोग भनेको के हो ?
2. हृदय तथा रक्त नली रोगको मुख्य कारणहरु के के छन् र जोखिम को कारकहरु बीचको आपसी अन्तर-समबन्ध के कस्ता छन् ?
3. कुन स्तरको शारीरिक गतिबिधि हप्तामा कति समय गर्न सिफारिस गरिएको छ ?
4. तरकारी तथा फलफूल दैनिक कति सर्भिङ्ग खान सिफारिस गरिन्छ ?
5. हृदय, रक्तनली तथा उक्त-रक्तचाप बढ्न बाट बच्ने उपाएहरु के के हुन् ?
