# Supplementary material for: Effect of peer-led health professional-supported intervention on cardiovascular disease risk reduction among industrial workers of Pokhara, Nepal: A quasi-experimental study
Source: PLOS Glob Public Health. 2025 Aug 12;5(8):e0004639. doi: 10.1371/journal.pgph.0004639 (PMC12342279; doi:10.1371/journal.pgph.0004639)
Supplement: S2 Text — (DOCX) [file pgph.0004639.s003.docx]

**Effect of Peer-Led Health Professional-Supported Intervention on Cardiovascular Disease Risk Reduction among Industrial Workers of Pokhara, Nepal: A Quasi-Experimental Study**

**S2 Brochure**

# हृदय तथा रक्तनली रोगको बारेमा जानकारी तथा यस्का जोखिमहरु बाट बच्ने उपायहरु सम्बन्धि सहयोगी पुस्तिका

## हृदय रोग भनेको के हो?

मुटु र रक्त नलीको रोग, जसलाई मुटु रोग पनि भनिन्छ, यसमा धेरै समस्याहरू समावेश छन्, जसमध्ये धेरै एथेरोस्क्लेरोसिससँग सम्बन्धित छन्। एथेरोस्क्लेरोसिस एक अवस्था हो जुन धमनीहरूको भित्तामा प्लाक भनिने पदार्थ निर्माण हुँदा विकसित हुन्छ। यो निर्माणले धमनीहरूलाई साँघुरो बनाउँछ, रगत प्रवाह गर्न गाह्रो बनाउँछ र अन्ततः यसले रगत प्रवाह रोक्न सक्छ। यसले हृदयघात वा स्ट्रोक निम्त्याउन सक्छ।

## रक्त नलिको संरचना

**धमनी:** अक्सिजन युक्त (शुद्ध) रगतलाई मुटुबाट शरीरका विभिन्न भागहरूमा लैजाने रक्तनली ।

**शिरा:** अक्सिजनको कमी भएको (अशुद्द) रगतलाई मुटुमा पुर्‍याउने रक्तनली |

**केशिका:** रगत र शरीरको तन्तुहरू बीच अक्सिजन, पोषक तत्वहरू, र अनावाश्यक उत्पादनहरूको आदानप्रदानको लागि धमनी र शिराहरू जोडिने ठाउ ।

## हृदय तथा रक्तनलिका रोगहरु

**कोरोनरी हृदय रोग:** मुटुको मांसपेशीमा आपूर्ति गर्ने रक्त वाहिकाहरूको रोग

**सेरेब्रोवास्कुलर रोग (मस्तिष्क पक्षघात) :** मस्तिष्कमा आपूर्ति गर्ने रक्त वाहिकाहरूको रोग

**परिधीय धमनी तथा गहिरो शिरा थ्रोम्बोसिस रोग:** हात र खुट्टाहरूमा आपूर्ति गर्ने रक्त वाहिकाहरूको रोग

**रूमेटिक हृदय रोग:** स्ट्रेप्टोकोकल ब्याक्टेरियाको कारणले लाग्ने रूमेटिक ज्वरोबाट मुटुको मांसपेशी र मुटुको भल्भमा क्षति भइ हुने हृदय रोग

**जन्मजात हृदय रोग:** जन्म दोषहरू जसले जन्मदेखि नै मुटुको संरचनाको विकृतिको कारणले गर्दा मुटुको सामान्य विकास र कार्यलाई असर गरेर हुने हृदय रोग

## हृदय रोगको मुख्य कराणहरु

- **उच्च रक्तचाप**
- **शारीरिक निष्क्रियता**
- **मोटोपना (तौल)**
- **खानपान**
- **धुम्रपान सेवन**
- रक्सीको हानिकारक प्रयोग
- चिनीरोग
- रगतमा लिपिडको मात्रा धेरै हुनु
- तनाव र
- वंशानुगत कारकहरू इत्यादि

## जोखिम को कारकहरु बीचको आपसी अन्तर-सम्बन्ध

**मोटोपना**

**शरीरको बोसोको मात्रा बढ्छ**

**उच्च रक्तचाप**

**एथेरोस्क्लेरोसिस**

**खानपान**

**न्यून शारीरिक गतिबिधि**

**धुम्रपान सेवन**

**हृदयघात, मस्तिष्क घात तथा अन्य जटिलताहरू समस्याहरू**

**हृदय तथा रक्त नली रोगको जोखिमहरुको बर्गिकरण**

| **हल्का जोखिमहरु** | **मध्यम जोखिमहरु** | **उच्च जोखिमहरु** |
| --- | --- | --- |
| **उमेर:** पुरुषहरूको लागि 40 भन्दा माथि, महिलाहरूको लागि 50 भन्दा बढी | **एक वा धेरै हल्का जोखिम कारकहरू संयुक्त:** उदाहरणका लागि, दुबै हल्का उच्च कोलेस्ट्रोल र हृदय रोगको पारिवारिक इतिहास भएको व्यक्तिलाई मध्यम जोखिम मानिनेछ \| | **धेरै मध्यम जोखिम कारकहरू संयुक्त:** उदाहरणका लागि, उच्च रक्तचाप, मोटोपन, र मधुमेह भएको व्यक्तिलाई उच्च जोखिम मानिनेछ। |
| **उच्च कम्मर परिधि:** पुरुषहरूको लागि 40 इन्च भन्दा बढी, महिलाहरूको लागि 35 इन्च भन्दा बढी | **अधिक तौल वा मोटोपना:** 25 भन्दा माथि BMI | **धुम्रपान:** चुरोट वा तंबाकूको प्रयोग |
| **प्रि-डायबिटिज:** रगतमा चिनीको मात्रा सामान्यभन्दा बढी हुनु तर मधुमेह मान्न पर्याप्त नहुनु | **उच्च रक्तचाप:** सिस्टोलिक **120-129 mmHg** को रिडिङ्ग वा डायस्टोलिक **80-89 mmHg** को रिडिङ्ग वा सो भन्दा माथि (औषधिले उपचार गरे पनि) | **मधुमेह:** इन्सुलिन उत्पादन वा उपयोगमा बाधाको कारणले उच्च रगतमा चिनीको मात्रा हुनु |
| **खानेकुरा:** तरकारी तथा फलफूलहरु दैनिक ५ सर्भिङ्ग भन्दा थोरै खाने |  |  |
| **शारीरिक व्यायाम:** एक हप्तामा कम्तिमा १५० मिनेट प्रति हप्ता मध्यम-तीव्रता शारीरिक गतिविधि वा ७५ मिनेट प्रति हप्ता कडा तीव्रता गतिविधि, वा दुवैको संयोजन नगर्ने |  |  |

## उच्च रक्तचाप

जब तपाईंको रक्त नालीहरूमा रगतको दबाव धेरै उच्च हुन्छ (140/90 mmHg वा उच्च) यस्तो अवस्थालाई उच्च रक्तचाप भनिन्छ। *उच्च रक्तचाप भएका व्यक्तिहरूले लक्षणहरू महसुस नगर्न सक्छन्। थाहा पाउने एक मात्र तरिका भनेको आफ्नो रक्तचाप जाँच गराउनु हो।*

## आफ्नो रक्तचाप नियमित जाच गराउनुहोस:

यदि तपाईंसँग हृदय रोगका लागि कुनै जोखिम कारकहरू छैनन् अथवा जोखिमहरू न्यून छन् भनेः कम्तिमा प्रत्येक वर्षमा एकपटक आफ्नो रक्तचाप जाँच गर्नुहोस्।

एक वा बढी जोखिम कारकहरू भएका वयस्कहरूका लागिः

**हल्का जोखिम कारकहरूः** प्रत्येक 6 महिनादेखि 1 वर्षसम्म सिफारिस गर्न सकिन्छ (जस्तै: उमेर, पारिवारिक इतिहास, उच्च कम्मर परिधि,प्रि-डायबिटिज, दैनिक ५ सर्भिङ्ग भन्दा कम तरकारी तथा फलफूल को प्रयोग, अपर्याप्त शारीरिक गतिबिधी )

**मध्यम जोखिम कारकहरूः** प्रत्येक 3 देखि 6 महिनामा सल्लाह दिन सकिन्छ (जस्तै: एक वा धेरै हल्का जोखिम कारकहरू संयुक्त, अधिक तौल वा मोटोपना, उच्च रक्तचाप)

**उच्च जोखिम कारकहरूः** गम्भीरताको आधारमा आवश्यकता अनुसार मासिक वा अझ बढी (जस्तै धेरै मध्यम जोखिम कारकहरू संयुक्त, धुम्रपान, मधुमेह)

**रक्तचापको वर्गीकरण**

| **रक्तचापको वर्गीकरण** | **सिस्टोलिक mm Hg (माथिल्लो मान)** |  | **डायस्टोलिक mm Hg**  **(तल्लो मान)** |
| --- | --- | --- | --- |
| सामान्य | १२० भन्दा कम | र | ८० भन्दा कम |
| केहि बढेको | १२०-१२९ | र | ८० भन्दा कम |
| पहिलो तह (Stage 1) | १३०-१३९ | वा | ८०-८९ |
| दोश्रो तह (Stage 2) | १४० वा धेरै | वा | ९० वा धेरै |
| उच्च रक्तचाप जोखिम  (Stage 3) | १८० भन्दा माथि | र/वा | १२० भन्दा माथि |

## बीएमआई (BMI)

BMI तौल-देखि-उचाइको एक सरल सूचकांक हो जुन सामान्यतया वयस्कहरूमा कम तौल, अधिक तौल र मोटोपनालाई वर्गीकृत गर्न प्रयोग गरिन्छ। यसलाई तौललाई किलोग्राममा र उचाइलाई वर्ग मिटरमा (किलोग्राम/मि^२^) विभाजित गरी परिभाषित गरिएको छ।

बीएमआई 18.5-24.9: सामान्य तौल

बीएमआई ≥ 25.0: अधिक तौल

बीएमआई ≥ 30.0: मोटापना

## शारीरिक गतिबिधि

शारीरिक गतिविधिलाई कंकाल मांसपेशीहरूद्वारा उत्पादित कुनै पनि शारीरिक गतिविधिहरु जसलाई ऊर्जा आवश्यक पर्छ

त्यस्तो शारीरिक गतिविधिहरु भनि विश्व स्वास्थ्य सङ्गठनले परिभाषित गरिएको छ |

शारीरिक गतिविधिहरु भन्नाले फुर्सदको समयमा, ठाउँहरूमा पुग्न र जानका लागि यातायातको प्रयोग, वा व्यक्तिको कामको भागको रूपमा गरिने सबै गतिविधिहरुलाइ जनाउँछ।

एक हप्तामा कम्तिमा **१५० मिनेट** प्रति हप्ता मध्यम-तीव्रता शारीरिक गतिविधि वा **७५ मिनेट** प्रति हप्ता कडा तीव्रता गतिविधि, वा दुवैको संयोजन ।

दैननक शारीरिक गतिबिधिको मात्रा मापनका लागि कम्तिमा १० मिनेटको मध्यम MET स्तरको गतिविधिको अवधि गणना गरिन्छ। तपाइँ आराम गर्दा भन्दा एक निस्चित समयावधिमा तपाईंले कुनैपनि काम गर्दा हने उर्जाको खर्चको मात्राको अनपुात लाइ Metabolic Equivalent of Work (MET) भनिन्छ | MET भनेको कुनै पनि काम गर्दा कति उर्जा खर्च हुन्छ भनेर वर्णन गर्ने एउटा तरिका हो | जस्तै कुनै गतिबिधिको MET मान ४ छ भने त्यसको अर्थ उक्त गतिबिधि गर्दा तपाइले आराम गर्दाको अवस्थामा भन्दा ४ गणुाले उर्जा प्रयोग गर्दै हनुहुन्छ भन्ने बुझिन्छ |

एक MET भन्नाले प्रति मिनेट प्रति किलोग्राम शरीरको तौलले लगभग ३.५ मिलिलिटर अक्सिजन गर्ने खपतलाइ बुझिन्छ । उदाहरणका लागि, यदि तपाईंको ७२.५ किलोग्राम तौल छ भने तपाईं आराममा हँदुा प्रति मिनेट लगभग २५४ मिलिलिटर अक्सिजन खपत गर्नुहुन्छ (७२.५ kg x 3.5 ml)| त्यस्तै उदाहरणका लागि, कपडा धुने कामको MET २.२ हन्छ, १० मिनेट कपडा धुदा २२ MET पुग्छ (२.२ * १०), सात दिन १० मिनेट कपडा धुने काम गर्नुहुन्छ भने १५४ MET पुग्छ |

१८ देन्ख ६५ वर्षका स्वस्थ वयस्कहरूका लागि, हप्तामा पाँच दिन ३० मिनेटको लागि मध्यम तीव्रता, वा हप्तामा तीन दिन २० मिनेटको लागि कडा तीव्रता शारीररक गतिबिधि सिफारिस गर्दछ । स्वस्थ वयस्कहरूलाइ सिफारिस गरिएको न्यूनतम ४५० देन्ख ७५० MET/min प्रति हप्ता खर्च गर्नुपर्छ ।

**ध्यान दिनु पर्ने कुरा:- *हृदयाघात भएका व्यक्तिले सुरुको दुई हप्तासम्म बढी शारीरिक परिश्रम गर्नु राम्रो हुँदैन तसर्थ मुटुरोग विशेषज्ञसँग परीक्षण गराई मुटुरोगको अवस्थाका बारेमा सही जानकारी लिएर उचित उपचार तथा सावधानीका उपायहरू अपनाउनु पर्छ |***

## तरकारी तथा फलफूल

दैनिक आहारको भागको रूपमा फलफूल र तरकारीहरू समावेश गर्नाले हृदय रोग तथा अन्य नसर्ने रोगहरुको जोखिम कम गर्न सक्छ। फलफूल र तरकारीहरूमा भिटामिन र खनिज, फाइबर जस्ता लाभकारी पोषक तत्वहरु पाइन्छ |

विश्व स्वास्थ्यसंघले प्रति दिन ४०० ग्राम भन्दा बढी फलफूल र तरकारीहरू उपभोग गर्न सुझाव दिएको छ | दैनिक एक बयास्कलाई कम्तिमा ५ सर्भिङ्ग (१ सर्भिङ्ग = ८० ग्राम) फलफूल, तरकारी अथवा दुवै मिलाएर खान सुझाब गरिन्छ |

| **मौसमी तरकारी** | **मौसमी फलफूल** |
| --- | --- |
| **जाडो मौसममा पाउने**   - साग - गाजर, मुला, सलगम, चुकंदर - फुलगोबी, बन्दागोबी, ब्रोकाउली - फर्सी | - सुन्तला, कागती, मौसम - अनार - श्याउ - केरा - अम्बा - अंगुर |
| **गर्मि तथा वर्षा मौसममा पाउने**   - केराउ - करेला - काँक्रा - सिमि - भिंडी - भान्टा - गोलभेडा - लौका, घिरौला | - स्ट्रबेरी - आप - मेवा - लिची - कटहर - खर्बुजा - भुइ-कटहर |

## धुम्रपानले कसरि मुटुमा असरहरु गर्छन

- मुटुको धड्कन र रक्तचाप बढाउछ
- रक्तनलीका पर्खालहरूलाई क्षति पुर्‍याउँछ
- रगत जम्ने प्रक्रिया बढाउछ
- मुटुमा पुग्ने अक्सिजनको मात्रा घटाउछ

## हृदय तथा रक्तनली रोगबाट बच्ने उपाएहरु

१. रक्तचाप नियमित जाच गराउनुहोस

२. हप्तामा कम्तीमा १५० मिनेट मध्यम तीव्रताको व्यायाम वा ७५ मिनेट उच्च तीव्रताको व्यायाम गर्ने लक्ष्य राख्नुहोस्

३. स्वस्थ तौल कायम राख्नुहोस्

४. कम्तिमा फलफूल र तरकारी दैनिक ५ सर्भिङ्ग खाने गर्नुहोस

५. धूम्रपान सेवन नगर्ने

## रक्तचाप कहिले कहिले नाप्ने

यदि तपाईंसँग हृदय रोगका लागि कुनै जोखिम कारकहरू छैनन् अथवा जोखिमहरू न्यून छन् भनेः कम्तिमा प्रत्येक वर्षमा एकपटक आफ्नो रक्तचाप जाँच गर्नुहोस्।

एक वा बढी जोखिम कारकहरू भएका वयस्कहरूका लागिः

- **हल्का जोखिम कारकहरूः** प्रत्येक 6 महिनादेखि 1 वर्षसम्म सिफारिस गर्न सकिन्छ
- **मध्यम जोखिम कारकहरूः** प्रत्येक 3 देखि 6 महिनामा चिकित्सकको सल्लाह अनुसार
- **उच्च जोखिम कारकहरूः** गम्भीरताको आधारमा आवश्यकता अथवा चिकित्सकको सल्लाह अनुसार मासिक वा अझ बढी

## हृदय, रक्तनली तथा उच्च रक्तचाप बाट बच्ने उपायहरु

1. **शारीरिक रूपमा सक्रिय हुनुहोस्** :- शारीरिक गतिविधिले तपाईंलाई स्वस्थ तौल र तपाईंको रक्तचाप कम गर्न मद्दत गर्न सक्छ।
2. **स्वस्थ तौल कायम राख्नुहोस्** :- १८ देखि २४ कि.ग्रा/मि२

- स्वस्थ आहार
- शारीरक गतिबिधि

1. **स्वस्थ आहार खानुहोस्** :- ताजा फलफूल र तरकारीहरू दैनिक ५ सर्भिङ्ग
2. **धुम्रपान नगर्नुहोस्** :-

- धूम्रपानको साटो कुनै मुखमा हाल्ने प्रतिस्थापक खानेकुरा छानौ *(जस्तै:-सुपारी, नरीवलका टुक्राहरु, ल्वागं, सुकमेल, चीवीगं गम, चकलेट )*
- दिमाग र हातलाई व्यस्त राख्नुहोस्
- आफूले धूम्रपान छाडदैछु भनेर बारम्बार साथीभाई अनि अरुलाई सुनाउने
- तलतल लाग्दा ब्रश गर्ने
- धुम्रपानलाइ नाइँ भन्ने
